# P31 Gut colonization of carbapenem-resistant Enterobacteriaceae among patients with haematological malignancies in National Cancer Institute, Sri Lanka

**DOI:** 10.1093/jacamr/dlac004.030

**Published:** 2022-02-16

**Authors:** Y. W. S. Suranadee, Y. Dissanayake, B. M. B. T. Dissanayake, H. R. Jyalatharachchi, S. Gamage, S. P. Gunasekara

**Affiliations:** Department of Medical Microbiology and Immunology, Faculty of Medicine, University of Colombo, Sri Lanka; National Cancer Institute, Sri Lanka; Department of Microbiology, University of Sri Jayewardenepura, Sri Lanka; Department of Medical Microbiology and Immunology, Faculty of Medicine, University of Colombo, Sri Lanka; Department of Medical Microbiology and Immunology, Faculty of Medicine, University of Colombo, Sri Lanka; National Cancer Institute, Sri Lanka

## Abstract

**Background:**

Carbapenem-resistant Enterobacteriaceae (CRE) are the most critical group of MDR bacteria that pose a threat to human health especially affecting patients with haematological malignancies. Knowledge on the prevalence of CRE colonization of the gut will help healthcare providers to be vigilant in-patient management. However, there are no local studies exploring the gut colonizing CRE among patients with haematological malignancies. Hence, this study aimed to determine the prevalence of CRE genes in colonizing CRE isolates in patients with haematological malignancies and evaluate their antibiotic susceptibility pattern.

**Methods:**

Rectal swab samples collected from patients with haematological malignancies were screened with selective chromogenic agar for CRE. Species identification and ABST of CRE isolates were done using VITEK® 2 system. *bla*_KPC_, *bla*_NDM_ and *bla*_OXA−48_ genes were identified using an in-house conventional multiplex PCR, with previously described method and primers.

**Results:**

A total of 264 adult patients with haematological malignancies were included in the study. Prevalence of gut colonization of CRE was 35.2% (93/264). A total of 119 CRE isolates from 93 study subjects were further studied. *Klebsiella pneumoniae* was the predominant carbapenemase producer [68/119 (57%)] among the isolates. Sixty-one (51.2%) isolates harboured *bla*_NDM_, 48 (40.3%) *bla*_OXA−48_ and 12 (10%) *bla*_KPC_. Three isolates (2.5%) were shown to harbour all three genes and 24 (20%) two genes. Twenty (16.8%) of isolates tested negative for all three genes.

**Conclusions:**

A relatively high prevalence of CRE gene occurrence and co-occurrence was detected in the study population. This indicates a potential challenge to infection prevention and control in the institute as colonization with CRE carries a threat to endogenous infection and cross transfer.

**Limitations:**

In-house conventional PCR method was used to identify CRE genes. Twenty isolates that were negative for all tested genes need to be studied further.

